# Of cells and tissues: Identifying the elements of a diabetic cardiac in vitro study model

**DOI:** 10.21203/rs.3.rs-5125697/v1

**Published:** 2024-12-20

**Authors:** Ivana Hernandez, C. Gobinath, Andie E. Padilla, Carla D. Loyola, Binata Joddar

**Affiliations:** The University of Texas at El Paso; The University of Texas at El Paso; The University of Texas at El Paso; The University of Texas at El Paso; The University of Texas at El Paso

**Keywords:** Cardiomyocytes, Contractility, Healthy cardiac tissue, Disease Pathology, Type-II Diabetes, Connexin, Myosin Heavy Chain

## Abstract

This study aimed to elucidate the impact of advanced glycation end products (AGEs) and glucose shock on cardiomyocyte viability, gene expression, cardiac biomarkers, and cardiac contractility. Firstly, AGEs were generated in-house, and their concentration was confirmed using absorbance measurements. AC16 cardiomyocytes were then exposed to varying doses of AGEs, resulting in dose-dependent decreases in cell viability. The maximum tolerated dose of AGEs was determined, revealing significant downregulation of the cardiac gene gap junction alpha 1 (GJA1). Furthermore, the study assessed the effects of AGEs, glucose shock, and their combination on biomarkers, cardiac myosin heavy chain (MHC), and connexin-43 (Cx-43) in AC16 cells. It was found that AGEs supplementation induced an increase in MHC expression while reducing Cx-43 expression, potentially contributing to cardiac dysfunction. Glucose shock also affected cardiomyocyte contractility, highlighting the complex interplay between AGEs, glucose levels, and cardiac function. Additionally, human iPSC-derived cardiomyocytes were subjected to varying doses of AGEs, revealing dose-dependent cytotoxicity and alterations in contractility. Immunostaining confirmed upregulation of MYH7, a cardiac gene associated with muscle contraction, in response to AGEs. However, the expression of Cx-43 was minimal in these cells. This comprehensive investigation sheds light on the intricate relationship between AGEs, glucose shock, and cardiomyocyte function, providing insights into potential mechanisms underlying cardiac dysfunction associated with metabolic disorders such as diabetic cardiomyopathy (DCM).

## 1. Introduction

Diabetes poses a significant global health challenge, with its complications leading to substantial morbidity and mortality [1, 2]. Elevated glucose levels affect various cell types differently, with those expressing high levels of the glucose transporter 1 (GLUT 1), like vascular endothelial cells, being particularly vulnerable to damage [1, 3]. Renal mesangial cells, for instance, undergo changes resembling the diabetic phenotype due to increased glucose uptake [1, 4]. The process involves the formation of advanced glycation end products (AGEs), where glucose forms covalent adducts with proteins, including structural proteins like collagen and functional ones like plasma proteins [1, 6]. These glycation reactions contribute significantly to diabetic complications such as nephropathy, retinopathy, and neuropathy [1, 6].

AGEs accumulation, both endogenously and from exogenous sources like food, disrupts normal protein function and structure, triggering cellular dysfunction and tissue damage [1, 6]. The reactions involved include the interaction of sugars with proteins, and progress through early, intermediate, and late stages, culminating in irreversible AGEs formation. Reactive intermediates like α-dicarbonyls play crucial roles in this process [1, 6]. AGEs are implicated in various age-related diseases, including diabetes, cataracts, atherosclerosis, nephropathy, and neurodegenerative disorders like Alzheimer’s disease as they can form through auto-oxidative pathways [1, 7]. Exogenous AGEs ingested through food further contribute to the AGEs burden in tissues. These AGEs can be classified into different types based on their cross-linking properties, all of which contribute to tissue damage and disease progression in diabetes [1, 8].

In the context of heart disease and cardiac function, a recently published study highlighted the association of AGEs with cardiac function in a large population-based cohort study [1, 9]. This study showed that AGEs accumulation was associated with prevalent heart failure and deterioration of cardiac systolic function [1, 9]. These findings as well as other such reports [1, 10], motivated us to perform further research into understanding the mechanisms linking AGEs to cardiac dysfunction on a cellular level. Outcomes can generate new knowledge on the impact of AGEs as a proposed therapeutic target in the treatment of heart failure.

Cardiovascular disease is known to involve cardiac cell dysfunction and the death of cardiomyocytes. Diabetic cardiomyopathy (DCM) is the main cause of heart failure in diabetic patients and one of the most lethal complications. Clinical hyperglycemia and chronic exposure to elevated blood glucose can generate AGEs. The presence of AGEs in cardiac tissues is known to cause cardiac cell death in the absence of myocardial ischemia. AGEs generated in the circulation of diabetic patients were reported to affect the function of the vascular wall [1, 11]. Although it has been elucidated from prior studies that cardiac fibroblasts are known to induce injury or death of cardiomyocytes in the pathogenesis of diseases such as cardiac hypertrophy, the relationships between the dysfunction or cell death of cardiomyocytes and AGEs remain unclear.

In a prior study by our group, we aimed to understand how hyperglycemia affects cardiomyocytes and contributes to cardiac fibrosis, a precursor to vascular complications. Cardiomyocytes were cultured under normal and hyperglycemic conditions, and RNA sequencing was used to identify differentially expressed genes (DEGs) related to inflammation and fibrosis. The results showed significant alterations in gene expression, including matrix metalloproteinases and inflammatory mediators, particularly under severe hyperglycemia. Protein expression of the YAP1/TAZ pathway, known for its involvement in fibrosis and inflammation, was also upregulated under severe hyperglycemia. Further the investigation revealed that inhibition of the ERK 1/2 signaling cascade reduced YAP1/TAZ expression, suggesting a potential therapeutic strategy to mitigate hyperglycemia-induced cardiovascular damage and inflammation [1, 12]. Building on existing work, in this study, we exposed human cardiomyocytes, AC16, and human induced pluripotent stem cell-derived cardiomyocytes (iCell cardiomyocytes) [1, 13] to an optimized dose of AGEs, or hyperglycemia to compare their effects on cardiac function. Next, we exposed these cardiomyocytes to treatments including AGEs and or hyperglycemia in the presence and absence of an AGEs inhibitor. The AGEs inhibitor is known to block the receptor for AGEs (RAGE) and prevent the supplemented AGEs from demonstrating its downstream effects [1, 14]. The effects of administering these varying biochemical stressors and inhibitors were assessed by studying the effects on the cardiac gap junction communication using Cx-43 expression in the AC16 cardiomyocytes. The expression of the cardiac MHC was also studied to measure the cardiac stress generated by varying biochemical treatments in both cell types. Next, the contractile human cardiomyocytes were exposed to varying biochemical shock treatments and their effects were evaluated by recording changes in spontaneous cardiac beating and electrophysiology. Results generated from this study will help develop a new model that can serve as an in-vitro tool for investigating cellular functional relationships relevant to DCM and future cardiac bioengineering applications.

## 2. Materials and Methods

### 2.1 Preparation of Advanced Glycation End Products (AGEs)

Advanced glycation end products (AGEs) are formed via non-enzymatic reactions between sugars and proteins [1, 15]. Methylglyoxal is a precursor for AGEs and contributes to various dysfunctions like diabetes and cancer. The AGEs used in this project were synthesized in-house following published procedures [1, 16]. The conjugation product formed between AGEs and BSA (10mg/ml) (Cat. No. 121800, Sigma-Aldrich, St. Louis, MO, USA) was used as a reference standard in this study. For the synthesis of AGEs-BSA, fatty acid-free BSA fraction V (20 mg/mL) (Sigma-Aldrich, St. Louis, MO, USA) was incubated with 0.5 mM glucose (Sigma-Aldrich, St. Louis, MO, USA) in 0.1 M potassium phosphate (Cat. No. P9791, Sigma-Aldrich, St. Louis, MO, USA) buffer containing 0.02% sodium azide (Cat. No. S2002, Sigma-Aldrich, St. Louis, MO, USA) and 1 mM diethylenetriamine penta-acetic acid (Cat. No. D1133, Sigma-Aldrich, St. Louis, MO, USA) all these were in a CO_2_ incubator (Vision Scientific Co., Daejeon, Republic of Korea) at 37°C for 8 to 10 weeks [1, 17]. Non-modified BSA, in the absence of sugar, was subjected to the same procedure incubated as described above and used as a control. The prepared AGEs were dialyzed for 24 h against potassium phosphate buffers to remove non-reactive sugars and other low-molecular-weight reactants. Then, the AGEs and the BSA control were measured and validated with a multi-microplate reader using an excitation/emission wavelength of 370 nm/440 nm (BioTek, SYNERGY H1 microplate Reader, Agilent Technologies, Santa Clara, CA). Absorbance values measured through spectrophotometry were correlated with AGEs product concentration using the principles of the Beer-Lambert law. From published reports of others, the mechanisms involved in AGEs formation is complex and can be initiated by a great variety of precursors leading to the generation of many chemically diverse AGEs molecules [1, 18].

### 2.2 Cardiac Cell Cultures

Two different types of human cardiomyocytes were used in this study. The first one was a non-contractile phenotype which has been extensively used in prior studies from our lab [1, 19]. The human cardiomyocyte AC16 cell line was purchased from Millipore (Cat. No. SCC109, Billerica, MA, USA) and maintained in Dulbecco’s Modified Eagle Medium (DMEM; Cat No.11965118, Invitrogen, Carlsbad, CA, USA) containing 10% FBS (Cat. No. A5670701, Gibco, Carlsbad, CA, USA), 100 U/mL penicillin and 100 mg/mL streptomycin (Cat. No:15140122, Invitrogen, MA, USA) and cells were cultured using either 5mM or 25mM D-glucose in a humified incubator at 37°C with 5% CO_2_ for at least 3 passages before being utilized for experiments.

The second cell type was a human stem cell derived contractile cardiomyocyte which is more suitable for electrophysiology investigations. Human induced pluripotent stem cell (iPSCs) derived cardiomyocytes (iCell cardiomyocytes^®^, Cat. No. R1105) were purchased from FUJIFILM Cellular Dynamics, WI, USA. These cells are mixtures of electrically active atrial, nodal, and ventricular-like myocytes. Before seeding these cells, 48-well tissue culture plates were coated with 0.1% gelatin at 37°C for at least 1 hour. Cardiomyocytes were initially plated at 600,000 cells per well according to manufacturer recommendation, using a plating medium and incubated at 37°C with 5% CO_2_ for 48 hours. After 48 hours post-plating, the plating medium was replaced with an appropriate volume of maintenance medium (Cat No. M1003, FUJIFILM Cellular Dynamics, WI, USA). The maintenance medium was replaced every other day for at least 14 days before treatment, as recommended by the manufacturer, allowing the cells to stabilize to in-vitro culture and passaging. For these cells, the plating and maintenance media are proprietary formulations and used in accordance with the vendor’s guidelines.

For measuring cardiac electrical activity, multielectrode array (MEA) plates were utilized. The MEA-6-well plate was coated with 40 µl of fibronectin solution (50µg/ml), prepared by diluting a stock solution of fibronectin (1mg/ml) in D-PBS and incubated in the cell culture incubator at 37°C for 1 h after which they were coated with 0.1% gelatin at 37°C for 1 hour to facilitate cell adhesion. Subsequently, iCell cardiomyocytes^®^ were plated onto the fibronectin-gelatin coated 6-well MEA plates. After aspirating the excess fibronectin solution, a cell suspension (40µl/well) containing approximately 97,000 plated cardiomyocytes was added over the recording electrode area. The plates were then incubated at 37ºC, 5% CO_2_ for 1 h to allow the cell attachment. Following cell attachment, a plating medium (Cat No. M1001, FUJIFILM Cellular Dynamics, WI, USA) was gently added to reach a final volume of 3 ml/well and plates were further incubated at 37°C, 5% CO_2_. The plating medium was then replaced with iCell cardiomyocytes maintenance medium (Cat No. M1003, FUJIFILM Cellular Dynamics, WI, USA) on day 2 post-plating. Thereafter, 50% spent medium was replaced with maintenance medium every 2–3 days. MEA recording was performed 10–14 days post-plating as recommended by the manufacturer using the Local Extracellular Action Potential (LEAP) assay protocol.

### 2.3 Optimization of the Dose of AGEs

AGEs solutions were made using a stock concentration of 500 µg/ml and diluted to constitute solutions representing 250 and 100 µg/ml respectively and the guidance for these concentrations in vitro studies were derived from prior published works by others [1, 20, 21]. Most of these studies were focused on investigating the effects of AGEs on HUVECs [1, 22]. These AGEs solutions were supplemented to stable in vitro culture passages of AC16 cardiomyocytes to evaluate their dose-dependent effects on the cell vitality after 24 hours and 48 hours of exposure. After 24- and 48-hours, the cells were washed and replenished with complete growth medium as described earlier. A live cell-staining assay using Calcein-AM (Live/Dead^®^ viability kits, Invitrogen, USA) was performed to assess the effects of the varying doses of AGEs shown above. The percentage of viable cells in each of these cultures was analyzed and reported as a percentage in comparison with the control cultures in which no AGEs treatment was added.

The Live cell detection technique (using Live/Dead^®^ viability kits, Cat. No. L3224, Invitrogen, USA) was adopted to test the viability of AC16 CMs after the treatment with AGEs (100µg/ml, 250µg/ml, 500µg/ml). Calcein-AM indicated live cells (green), and the viability of the cells was calculated by taking the total number of live cells in the in the AGEs (Advanced Glycation End Products) wells, dividing it by the total number of live cells in the controls wells and then multiplying the result by 100. The number of cells were quantified using ImageJ software.

The Live/Dead assay (using Live/Dead^®^ viability kits, Cat. No. L3224, Invitrogen, USA) was performed to assess the viability of iCell cardiomyocytes^®^ after the treatment with AGEs (100µg/ml, 250µg/ml, 500µg/ml). Calcein-AM indicated live cells (green) while the ethidium homodimers indicated dead cells (red). The % viability of the cells versus % of the dead cells was determined by taking the number of live or dead cells, dividing it by the total number of cells (which is the sum of live and dead cells) and then multiplying the result by 100, number of cells were quantified using Image J software.

### 2.4 Preparation of Varying Biochemical Shock Treatments

Five different solutions were prepared for the shock treatments. For preparing these solutions, mainly in-house prepared AGEs, D-Glucose (Cat. No. G7021, Sigma-Aldrich, St. Louis, Missouri, USA), and the AGEs inhibitor, FPS-ZM1 (Sigma) were used. AGEs solutions were made using a stock concentration of 500 µg/ml and diluted to constitute solutions representing 250 and 100 µg/ml. The glucose solution constituted a 50 mM solution of D-glucose that represented hyperglycemic exposure as demonstrated by our prior published works [1, 12]. The AGEs inhibitor, FPS-ZM1 is a high-affinity inhibitor of the receptor for advanced glycation end products (RAGE) [1, 23] and it was constituted to represent 0.2 mM concentration solution for supplementation. All solutions were syringe filtered using a 0.22 µm syringe filter (cat No. SLMPR25SS, Millipore/Sigma) for sterilization before being supplemented to cell cultures. It is to be noted that the AGEs inhibitor (FPS-ZM1) was added at least 30 min prior to the addition of other treatments to the selected sample sets during the experiments.

The five different solutions were the following, 1) AGEs (500 µg/ml); 2) D-Glucose (50 mM); 3) AGEs + D-Glucose; 4) FPS-ZM1 (0.2 mM) + AGEs and 5) FPS-ZM1 (0.2 mM) + AGEs + D-Glucose. Controls did not receive any of these treatments.

For the AC16 CM, treatments were provided for 24-hours up to 48 hours to study their effects on cells. For the iCell cardiomyocytes, treatments were only provided for 30 mins to study their effects on the cells. These time points were determined based on experiments performed earlier in this project as explained below.

For AC16 cardiomyocytes, which are very stable cell lines, treatments were administered for 24 to 48 hours to investigate the chronic or sustained effects on the cells. This extended duration allowed for the observation of slower cellular processes such as apoptosis, and long-term signaling pathway activation, which may not be evident with shorter exposure times in these cell types. The longer treatment period was also necessary for the accumulation of compounds within the cells, facilitating the study of dose-response relationships and metabolic changes.

In contrast, iCell cardiomyocytes, which are primary cell types, were treated for only 30 minutes to examine the acute or immediate effects of treatments. This brief exposure was ideal for capturing rapid cellular responses, which occur quickly and transiently. The short treatment duration minimized secondary changes, providing precise control over experimental conditions, and isolating the primary effects of the treatments. The layout of experiments using both cell types is summarized in Table 1 below.

### 2.5 Quantitative PCR (qPCR) Analysis

For this procedure, 50 ng of total RNA was converted to cDNA using the First Strand cDNA Synthesis Kit from OriGene Technologies, Inc., in a 20 µL reaction following the manufacturer’s instructions. The resulting cDNA was quantified using a NanoDrop OneC spectrophotometer from ThermoFisher Scientific, and absorbance ratios at 260/280 nm and 260/230 nm were recorded. RT-qPCR reactions were conducted on a Quantstudio 3 system from Applied Biosystems, Invitrogen. All samples including controls and experimental samples: 1) AGEs (500 µg/ml); 2) D-Glucose (50 mM); 3) AGEs + D-Glucose; 4) FPS-ZM1 (0.2 mM) + AGEs and 5) FPS-ZM1 (0.2 mM) + AGEs + D-Glucose, were run in triplicate using qPCR Tubes, 8 strips, 0.2 mL, with optical strips caps from Pure AMP PCR plastics, MTC^™^ Bio Incorporated, in a 20 µL reaction volume. Each reaction contained 4000 ng of cDNA, 10 µL of Go Taq qPCR Master Mix SYBR, 0.2 µL of supplemental CXR Ref. Dye from Promega, 1 µL of 10 mM primer mix (Fw, Rv) from Origene Tech Inc., and nuclease-free water. The primer sequences corresponding to the genes for evaluation are shown in Table 2 below.

To prevent contamination and primer-dimer formation, a no template control was included. The reaction protocol consisted of an initial denaturation step at 95°C for 10 minutes, followed by 40 cycles of 95°C for 15 seconds and 60°C for 15 seconds as per the Origene protocol. Quantification cycle (Ct) values were automatically calculated by the Quantstudio 3 software. Gene expression was evaluated using the comparative Ct method (2^−∆∆CT^), with average ∆CT values of GAPDH serving as the endogenous control to assess the stability of target gene expression. We adopted the individual efficiency corrected calculation method, which represents an enhancement over the commonly utilized 2^−ΔΔCT^ method. This method computes an individual efficiency for each sample, thereby averting potential issues associated with estimating background fluorescence. Consequently, this novel approach ensures greater accuracy in relative gene expression quantification [1, 24].

### 2.6 Immunostaining

Immunohistochemical analysis was performed to detect connexion-43 (Cx-43) and myosin heavy chain (MHC) in the cardiac cells, used in this study. To do this, cells were rinsed in 1× D-PBS and subsequently fixed in methanol for 15 min. For Cx-43 immunostaining, the cells were blocked for 1 h at room temperature, washed once, and incubated with the primary antibodies Cx-43 (1:400, Catalog: 3D8A5, Thermo Fisher Scientific, Waltham, MA) overnight at 4°C. The cells were then washed three times and probed with secondary antibodies, Alexa Fluor 647 goat antirabbit IgG (1:2000; Thermo Fisher Scientific, Waltham, MA), and incubated at room temperature for an hour. For the MHC immunostaining, cells were incubated with Cardiac myosin heavy chain primary antibodies (1:400, Catalog: MA-26180, Thermo Fisher Scientific, Waltham, MA) overnight at 4°C. After incubation, the cells were washed three times and probed with secondary antibodies Alexa Fluor 488 goat antimouse IgG (1:2000; Thermo Fisher Scientific, Waltham, MA), and incubated at room temperature for an hour. After 3 washes with D-PBS, the cells were stained with DAPI-Fluoromount for 3 min and mounted on glass slides using Fluoromount G mounting media (Electron Microscopy Sciences, Fort Washington, PA). The images were acquired using a Zeiss Axio observer A1 fluorescent microscope (Carl Zeiss Microscopy, Germany).

The ImageJ software (NIH) was used for image analysis [1, 25]. The analysis involved splitting the DAPI (blue) and Cx-43 (red) or MHC (green) channels to create separate images for each channel. The Cx-43 or MHC image was then further processed. To remove background noise, the “Subtract Background” function from the process menu was utilized. The next step involved selecting the “Histogram” option from the Analyze menu to obtain a mean intensity value. This value is automatically calculated from the distribution of gray values in the active image. The mean intensity for each Cx-43 or MHC expression was measured from three different images for each condition. The fluorescence of each of these biomarkers was presented as the mean fluorescence intensity which was calculated relative to the DAPI signal in any sample well for every image acquired. These mean intensities were plotted with reference to the control sample.

In this study, the expression of the Receptor for Advanced Glycation End (RAGE) products was studied since the involvement of RAGE signaling has been implied in diabetic disease pathologies in many bodily tissues and organs [1, 26]. For the RAGE expression analysis, AC16 CM cells were cultured in 6-well plates until they reached the confluency, followed by treatments with different biochemical conditions as described earlier. Cells were then permeabilized using Triton^™^ X-100 used at 0.1% (v/v, in PBS) and then incubated with RAGE primary antibodies (1:100; cat. No bsm 52809R, Bioss; Massachusetts, USA) overnight at 4°C. After incubation with the primary antibody, the cells were washed three times and probed with goat anti-rabbit secondary antibodies IgG (H + L), Alexa Fluor^™^ 488 (1:500; Thermo Fisher Scientific, Waltham, MA), and incubated at room temperature for an hour. After 3 washes with D-PBS, the cells were counterstained with DAPI Fluoromount-G^®^ (Cat. No. 0100 − 20; Southern Biotech, Birmingham, AL, USA) for 3 minutes and mounted using Fluoromount -G^®^ media (Cat. No. 0100–01; Southern Biotech, Birmingham, AL, USA). The images were acquired using a Zeiss Axio observer A1 fluorescent microscope (Carl Zeiss Microscopy, Germany). The normalized mean fluorescent intensity of RAGE was calculated relative to the DAPI signal in any sample well for every image acquired using Image J software. Images were acquired in triplicate for each sample and each well.

### 2.7 Time-Lapse Imaging to Monitor Cardiac Contractility

Time-lapse images of beating cardiac cells (iCell cardiomyocytes) in the presence and absence of varying biochemical treatments in a 6-well plate were captured using an ImageXpress XL fitted with an environmental chamber (37°C and 5% CO_2_). Sequential transmitted light images were captured with a 10× Leica Thunder Imaging Live Cell and 3D Assay fluorescent microscope (Leica Microsystems, Buffalo Grove, IL). Images were acquired at a frame rate of approximately 35 fps. The wells included 1) AGEs (500 µg/ml); 2) D-Glucose (50 mM); 3) AGEs + D-Glucose; 4) FPS-ZM1 (0.2 mM) + AGEs and 5) FPS-ZM1 (0.2 mM) + AGEs + D-Glucose and 6) Controls.

### 2.8 Electrophysiological Characterization of Cardiac Cultures

The LEAP assay from the Axion Maestro Edge system (Axion Biosystems, Atlanta, GA), an MEA-based recording device allowed us to study the extracellular action potential and high-resolution contractility waveforms in the iCell cardiomyocyte networks in response to varying biochemical treatments. The treatments included 1) AGEs (500 µg/ml); 2) D-Glucose (50 mM); 3) AGEs + D-Glucose; 4) FPS-ZM1 (0.2 mM) + AGEs and 5) FPS-ZM1 (0.2 mM) + AGEs + D-Glucose and 6) Controls. This technique allowed us to study an entire 6-MEA well with each well representing its own unique cell culture and conditions, allowing up to 6 experiments on one plate. Furthermore, treatments were subjected to each well for only 30 mins after which washouts were performed and LEAP activity was recorded in each well.

The outcomes from this experiment generated cardiac field potential and corresponding heat maps in response to cardiac contractile activity in the wells in the absence and presence of varying biochemical treatments. The extracellular action potential of each active electrode is color-coded: white/red represents high firing action potential. Blue/black represents low firing action potential. Each circle represents an active electrode within the MEA plate. Furthermore, the raw data was processed using the Axion Maestro software to yield beats per minute or cardiac contractility in the wells in the absence and presence of varying biochemical treatments.

### 2.9 Statistical Analysis

To determine statistical significance, a one-way ANOVA was conducted, followed by a post-hoc Tukey’s multiple comparison test. A p-value of less than 0.05 was considered statistically significant and denoted by an asterisk (*). If the p-value was higher than this, it was deemed statistically non-significant and denoted by “ns.” The number of technical replicates included was n = 3, and each experiment was repeated twice for reproducibility unless otherwise mentioned.

## 3. Results

### 3.1 Generation and Confirmation of AGEs production

Using a microplate reader, in-house prepared AGEs samples were first added in triplicates to 96-well plates and their absorbance was measured. The absorbance values were used to calculate the concentration of the AGES generated in-house considering the values of absorbance of the AGES-BSA product as a reference. The in-house synthesized AGES product concentration was found to vary between 13.1–14.28 mg/ml, and 13.8 mg/ml and was used as a stock treatment for cell experiments in this study.

### 3.2 AC16 CM Cell Viability and Gene Expression Alterationin Response to Varying Doses of AGEs

**Figure 1:**
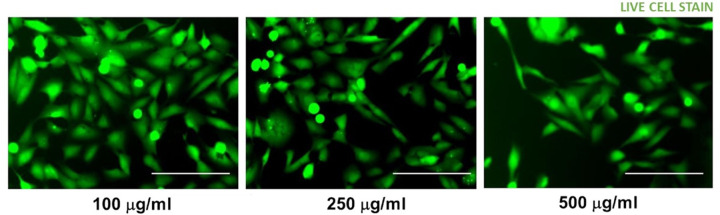
Dose dependent effects of AGEs on AC16 cardiomyocytes. The varying doses applied included 100 µg, 250 and 500 µg for 24 hours after which the cells were washed and replenished with complete growth medium. A live cell staining assay was performed to assess the effects of the varying doses of AGEs shown above. Scale bar indicates 100 µm.

Varying doses of AGEs were applied to AC16 cardiomyocytes for a duration of 24 hours in a sequence of doses including 100 µg/ml, 250 µg/ml, and 500 µg/ml, respectively. Following the exposure period, the cells were washed and provided with a complete growth medium. Subsequently, a live cell staining assay was conducted to evaluate the impact of the different doses of AGEs on the cells. Table 3 summarizes the percentage of living cells related to control after 24-hours. With increasing doses of AGEs administered, the cell viability was decreased proportionately with approximately 77%, 65%, and 44% of live cells observed in 100 µg/ml, 250 µg/ml, and 500 µg/ml, respectively, as shown in Fig. 1. From these results, the lethal concentration approximating 50% (LC50) is 500 µg/mL, as it caused death in about 50% of the cell population under specified conditions.

To determine the maximum tolerated dose of AGEs by the AC16 cardiomyocytes, 500 µg/ml of AGEs was administered to the cell cultures for a total 48-hour exposure period after which their total RNA was extracted and gene expressions analyzed for GAPDH and GJA1.

Utilizing the individual efficiency corrected calculation method, an enhancement over the commonly utilized 2^−ΔΔCT^ method, we calculated the average ΔCT of the target genes, GJA1 calculated from at least three data points recorded per sample extract. Next, the average ΔCT of the reference of housekeeping gene, GAPDH was calculated from at least three data points recorded. Following this, the expression fold change for gene expression of the target genes was calculated using the 2^−ΔΔCT^ method. Then these values were normalized to the control sample values calculated similarly using the steps enlisted above. It was assumed that the gene expression levels for both target and reference genes would remain relatively stable in the absence of any treatment in the controls. This led to the control sample values depicting expression fold change for gene expression of the target genes equaling ‘one.’ The experimental samples were included alongside the controls showing fold change expression in comparison with the controls.

**Figure 2:**
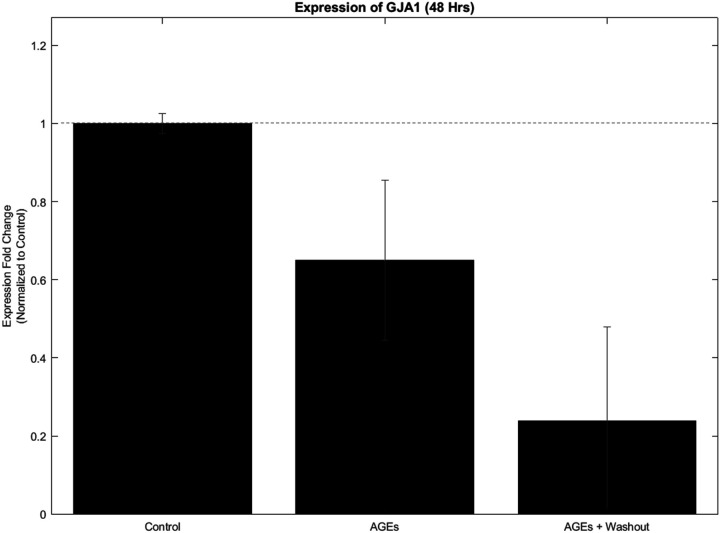
Dose dependent effects of AGEs on AC16 cardiomyocytes. The varying doses applied included 100 µg, 250 and 500 µg for 24 hours after which the cells were washed and replenished with complete growth medium. A live cell staining assay was performed to assess the effects of the varying doses of AGEs shown above. Scale bar indicates 100 µm.

Shown in Fig. 2 are relative gene expression changes for GJA1 normalized to GAPDH for the sample that they were extracted from including AGES (500 µg/ml for 48 hours), post-washouts, and controls that did not receive any AGEs. Outcomes indicate that the administration of AGEs (500 µg/ml) for 48 hours to AC16 CM cultures significantly downregulated their expression of the cardiac gene, GJA1 that was not restored post washout of the AGEs from the cultures. This helped determine the maximum toxicity dose and exposure period for AGEs in our study, using a robust human cardiac cell phenotype.

#### 1. Differential Protein and Gene Expression of Cardiac Biomarkers with Varying Biochemical ShockTreatments in AC16 CMs

In response to the varying treatments that included AGEs (500 µg/ml); D-Glucose (50 mM); AGEs + D-Glucose; FPS-ZM1 (0.2 mM) + AGEs and FPS-ZM1 (0.2 mM) + AGEs + D-Glucose, the expression of cardiac MHC and Cx-43 was confirmed via immunostaining. Controls did not receive any of these treatments. While it is widely believed that Cx-43 helps with electrical coupling and signal transduction in the ventricular myocardium, the cardiac MHC functions as a stress filament in cardiac cells and is helpful in maintaining cell shape and mechanical integrity. In cellular biology, the load or mechanical stress on a cell can be distributed between different types of cytoskeletal filaments such as Cx-43 and cardiac MHC. Therefore, studying the varying expressions of both cardiac biomarkers is important to assess the effects of biochemical stressors on cardiac function. The distribution of the total load on a single cell is evenly shared by cellular stress fibers such as the cardiac MHC and by the cardiac gap junction, Cx-43, under healthy conditions. Imbalance in this complex supramolecular arrangement of protein components indicates development of a diseased state [1, 27].

**Figure 3:**
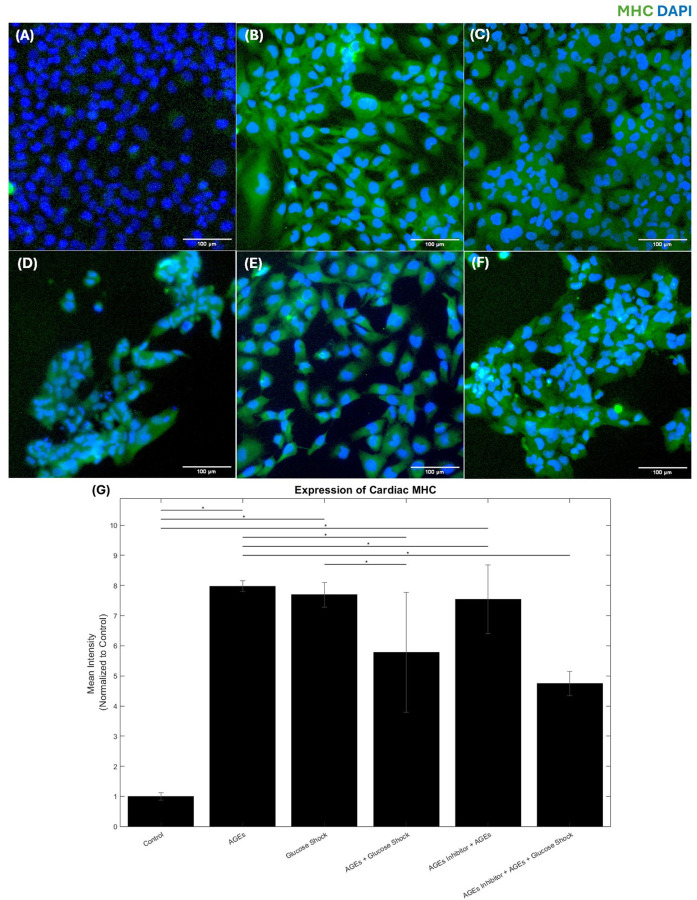
AC16 Cardiomyocytes Cardiac Myosin Heavy Chain (MHC) immunostaining for different experimental conditions: (a) Control, (B) AGEs, (C) Glucose Shock (50 mM), (D) AGEs followed by Glucose Shock, (E) Inhibitor followed by AGEs, (F) Inhibitor followed by AGEs and Glucose Shock. (G) Shows the expression levels of cardiac MHC calculated, data is represented as a normalized mean intensity to control. * Represents p<0.05.

As shown in Fig. 3, the expression of the cardiac MHC was significantly enhanced in the cultures that received treatments for a 24-hr exposure period (p < 0.05) in comparison with controls. The maximum increase of the cardiac MHC expression was observed in the cultures that received AGEs (500 µg/ml) and was not restored by the addition of the AGEs inhibitor. The samples that received glucose shock also showed an enhanced expression of the cardiac MHC. A combination of both AGEs and glucose also showed an enhanced expression of cardiac MHC and the pre-administration of the AGEs inhibitor helped regulate this scenario in the respective sample wells. The results from Fig. 3 also confirm that the enhanced expression of the cardiac MHC is related to cardiac stress generation as also stated by numerous reports in this field [1, 28].

**Figure 4:**
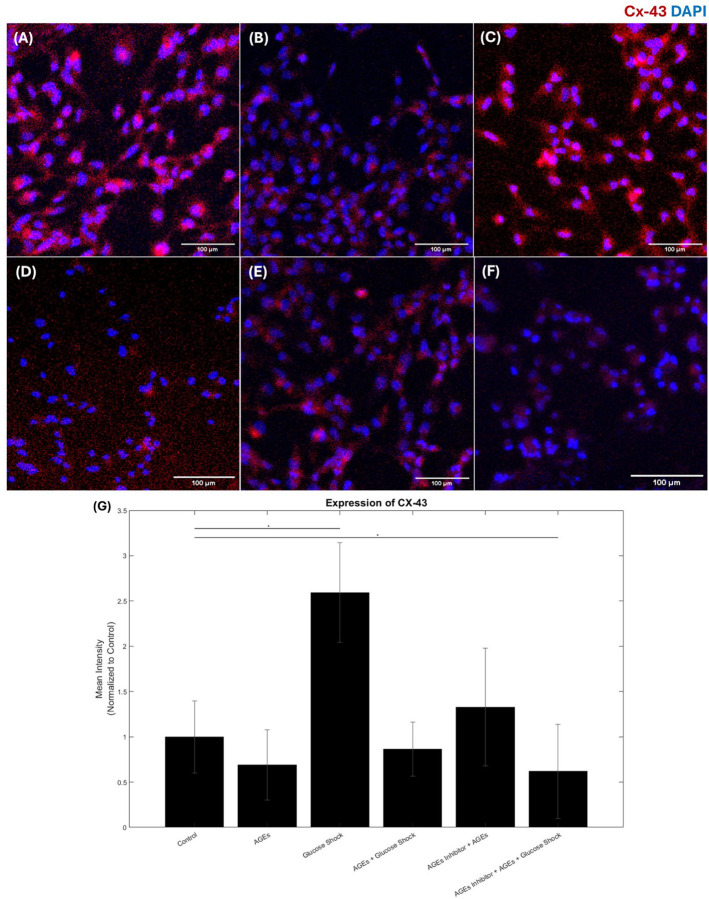
AC16 Cardiomyocytes Connexin 43 (Cx-43) immunostaining for different experimental conditions: (A) Control, (B) AGEs, (C) Glucose Shock (50 mM), (D) AGEs followed by Glucose Shock, (E) Inhibitor followed by AGEs, (F) Inhibitor followed by AGEs and Glucose Shock. (G) Shows the expression levels of Cx-43 calculated, data is represented as a normalized relative mean intensity to control. * Represents p<0.05.

As shown in Fig. 4, the expression of the cardiac Cx-43 was significantly diminished in the cultures that received treatments for a 24-hour exposure period (p < 0.05) in comparison with controls. The maximum reduction of the Cx-43 expression was observed in the cultures that received AGEs (500 g/ml) only or AGEs with other treatments. The pre-administration of the AGEs inhibitor helped restore the downregulated expression of Cx-43 in the samples that received AGEs-based treatments. These outcomes confirm that the reduced expression of the Cx-43 is related to significantly reduced levels of the principal gap junction protein, Cx-43, potentially contributing to cardiac dysfunction followed by cardiac stress generation [1, 29]. However, an enhanced Cx-43 expression was noted in samples treated with hyperglycemic shock and is a unique finding from our study.

**Figure 5:**
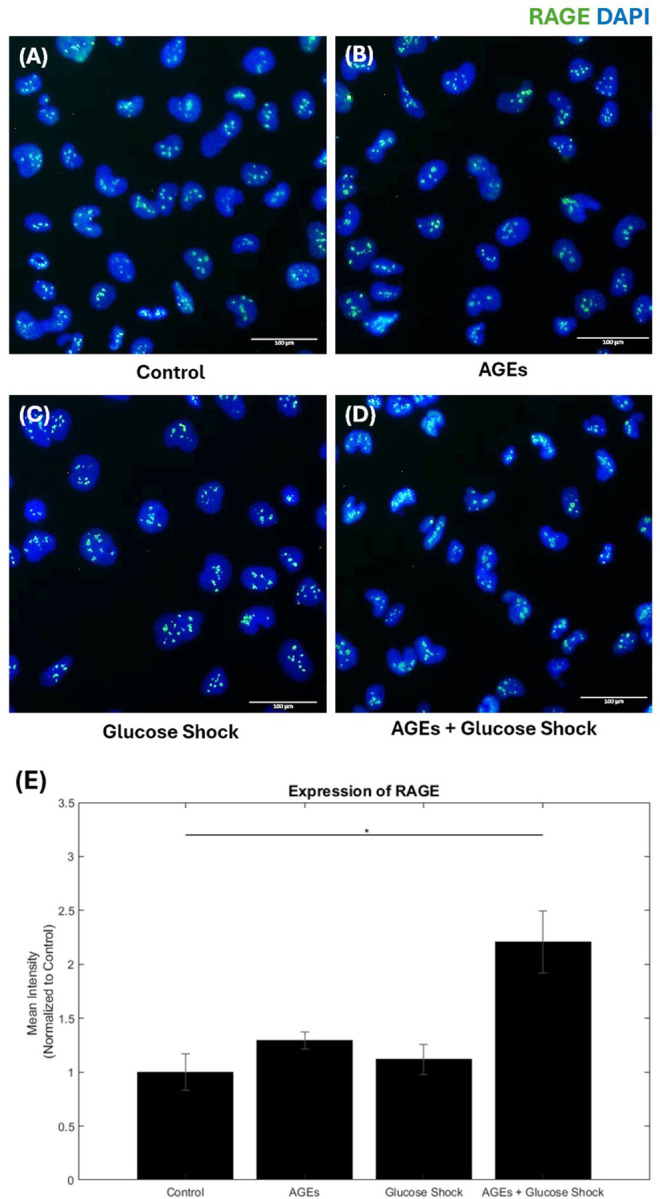
AC16 Cardiomyocytes RAGE expression for different experimental conditions: (A) Control, (B) AGEs (500µg/ml), (C) Glucose Shock (50 mM), (D) AGEs followed by Glucose Shock, (E) Shows the expression levels of RAGE calculated, data is represented as a normalized relative mean intensity to DAPI. * Represents p<0.05.

Shown in the Fig. 5 are the immunostaining results and RAGE expression alongside the quantification of mean fluorescence intensity relative to the DAPI signal in AC16 cells treated with AGEs (500µg/ml), D-Glucose (50mM), AGEs + D-Glucose for 24 hours. Control cells did not receive any treatments.

Compared to the control group, treatment with AGEs + D-Glucose showed a significantly altered RAGE expression in AC16 Cardiomyocytes. Treatments with AGEs or D-Glucose alone slightly upregulated the RAGE expression. AGEs + D-Glucose in combination resulted in significant upregulation. These results suggest that RAGE expression was upregulated in response to AGEs and glucose shock due to hyperglycemic conditions and inflammation.

Supplementary Fig. 1 shows the results of RAGE expression in cells treated with FPS-ZM1 (AGEs inhibitor) + AGEs and FPS-ZM1 + AGEs + D-Glucose. The addition of FPS-ZM1 inhibitor with AGEs modified the RAGE expression profile, and the combining FPS-ZM1, AGEs and D-glucose also altered the RAGE expression.

**Figure 6:**
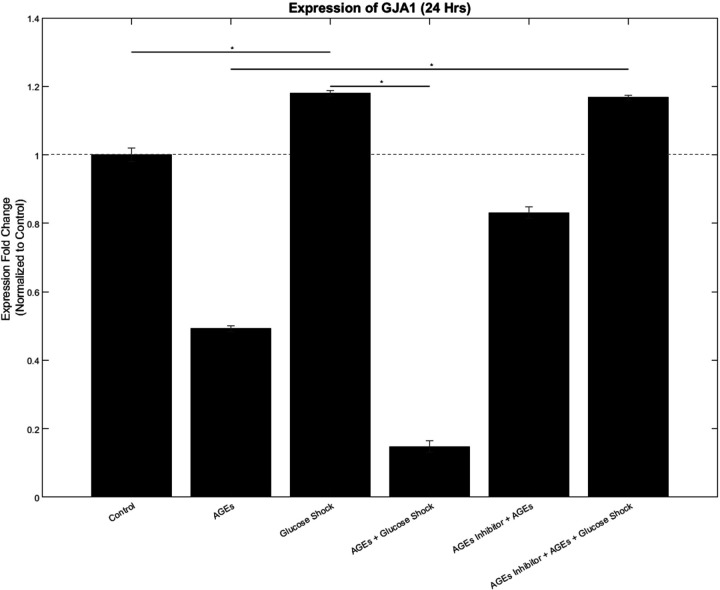
Expression Fold Change for gene expression of Cx-43 (encoded by GJA1) in AC16 cardiomyocytes under different experimental conditions after 24 hours of exposure. Data is represented as a normalized relative fold change to control. * Represents p<0.05.

Shown in Fig. 6 are relative gene expression changes for GJA1 normalized to GAPDH for the sample that they were extracted from including AGEs (500 µg/ml); D-Glucose (50 mM); AGEs + D-Glucose; FPS-ZM1 (0.2 mM) + AGEs and FPS-ZM1 (0.2 mM) + AGEs + D-Glucose, for 24 hours. Controls did not receive any of the above treatments. Outcomes indicate that the administration of AGES, AGEs + D-Glucose, FPS-ZM1 + AGEs, and FPS-ZM1 + AGEs + D-Glucose to AC16 CM cultures significantly altered their expression of the cardiac gene, GJA1.

### 3.4 Studying the impact of varying doses of AGEs on iCell cardiomyocyte cell viability and gene expression, alongside examining the alteration in cardiac biomarkers’ protein and gene expression under different biochemical shock treatments

**Figure 7:**
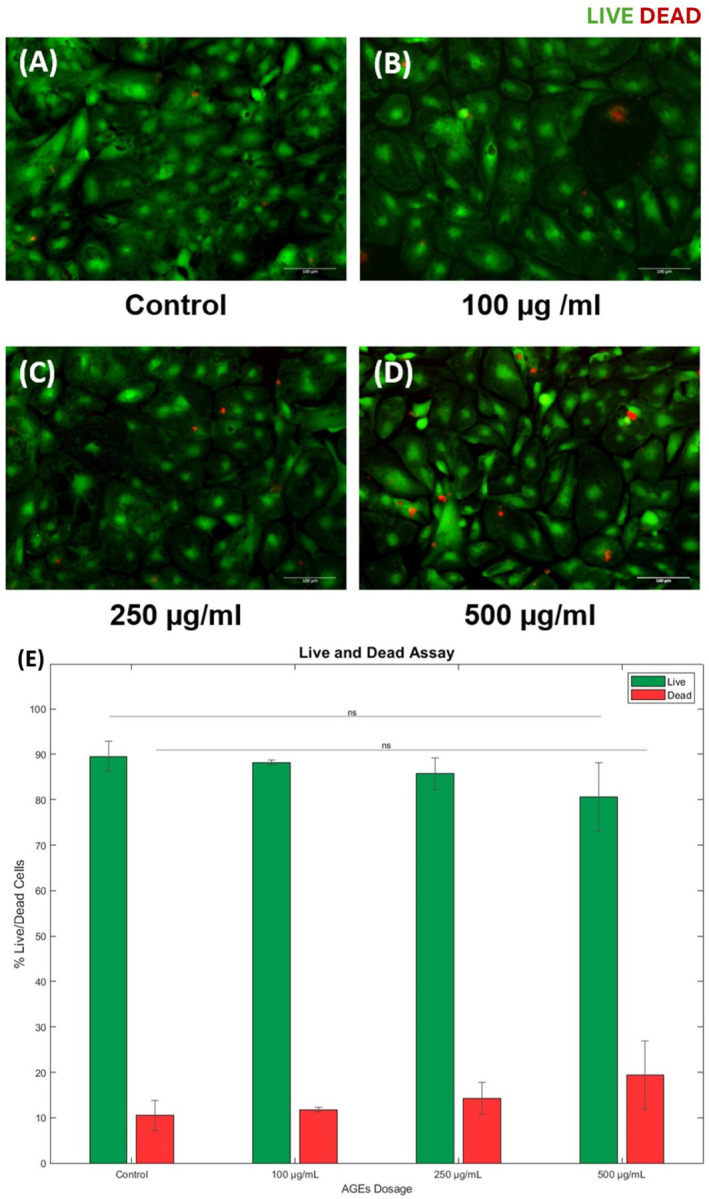
Dose dependent effects of AGEs on iCell Cardiomyocytes. (A) The varying doses applied included 100 µg, 250 and 500 µg for 30 mins after which the cells were washed and replenished with complete growth medium. (B) Shows the percentage cell viability measured from the live dead assay performed to assess the effects of the varying doses of AGEs.

As shown in Fig. 7, a live/dead assay was performed to assess the cytotoxicity of varying doses of AGEs on iCell cardiomyocytes. Cells were treated with varying concentrations of AGEs (100µg/ml, 250 µg/ml, 500 µg/ml) for 30 minutes, followed by washing and placed in the complete growth medium. Results revealed a dose-dependent trend in cell viability. The maximum cell viability and relatively minimum cell death were noted at 100 µg/ml compared to the untreated control. However, at 250 µg/ml, there was a noticeable increase in cell death compared to the lower dose, and at 500µg/ml, significant cell death occurred compared to the lower doses. Specifically, the cell viability decreased to 88 ± 0.483%, 85 ± 3.47%, and 80 ± 7.57%, with a notable increase in the percentage of cell death observed at 500 µg/ml, as shown. Based on these results, the 500 µg/ml AGEs were not supplemented to these cells for further experiments.

**Figure 8:**
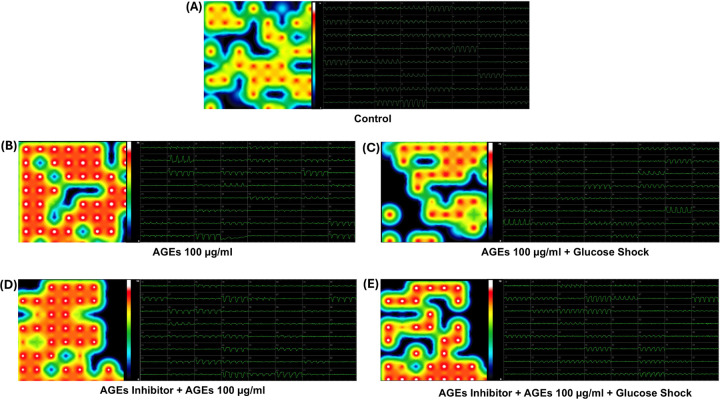
Beat Rate heat maps and waveforms studied in relation with dose dependent effects of AGEs on iCell Cardiomyocytes. Image shows applied AGEs at a 100 µg/ml concentration for 30 mins after which their contractility characterization was performed. Controls included wells with cells that did not receive any treatments and were washed and replenished with complete growth medium. The figure labels are as follows: (A) Control (Scale: 0 to 76), (B) AGEs (Scale: 0 to 56), (C) AGEs + Glucose Shock (Scale: 0 to 70), (D) AGEs Inhibitor + AGEs (Scale: 0 to 56), and (E) AGEs Inhibitor + AGEs 100 µg/ml + Glucose Shock (Scale: 0 to 70).

**Figure 9:**
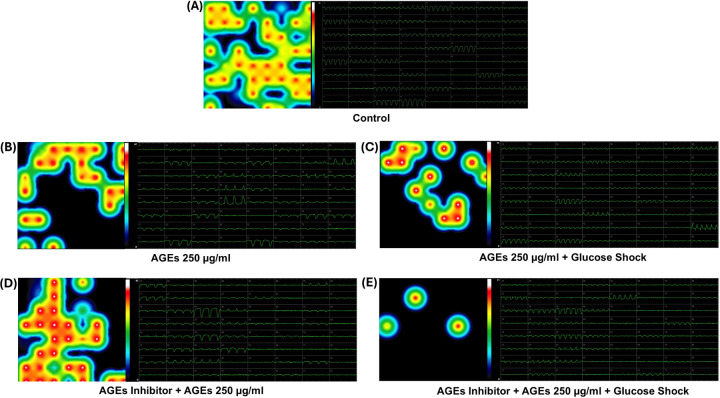
Beat Rate heat maps and waveforms studied in relation with dose dependent effects of AGEs on iCell Cardiomyocytes. Image shows applied AGEs at a 100 µg/ml concentration for 30 mins after which their contractility characterization was performed. Controls included wells with cells that did not receive any treatments and were washed and replenished with complete growth medium. The figure labels are as follows: (A) Control (Scale: 0 to 76), (B) AGEs (Scale: 0 to 56), (C) AGEs + Glucose Shock (Scale: 0 to 70), (D) AGEs Inhibitor + AGEs (Scale: 0 to 56), and (E) AGEs Inhibitor + AGEs 100 µg/ml + Glucose Shock (Scale: 0 to 70).

Shown in Fig. 8 and Fig. 9 are cardiac beat rate heat maps and waveforms studied in relation to dose dependent effect of AGEs on iCell cardiomyocytes. The varying doses of AGEs applied included 100µg/ml and 250µg/ml each for 30 mins after which their contractility was assessed. Also studied were samples that received varying doses of AGEs + Glucose in the presence and absence of the AGEs inhibitor. Controls included wells with cells that did not receive any treatments and were washed and replenished with a complete growth medium.

Supplementary Fig. 2 illustrates the impact of glucose shock on iCell Cardiomyocytes, with a concentration of 50 mM D-Glucose for 24 hours resulting in a significant increase in beat rate compared to controls. This highlights the effect of glucose shock on cardiomyocyte function. Supplementary Fig. 3 examines cardiac contractile behavior under various conditions, revealing stable beat rates around 60 BPM in both control and AGEs-treated groups. However, glucose shock led to an increase in beat rate to 72 BPM during treatment, followed by a decrease to 50 BPM post-wash, indicating a transient effect of glucose shock on cardiomyocyte contractility. In contrast, when the experimental conditions involved not only a high concentration of AGEs (500 µg/ml) but also the presence of an inhibitor and/or glucose shock, the cardiomyocytes ceased beating entirely. Unlike the AGEs and glucose shock group, these cells did not recover their beating activity even after the post-wash period. This suggests that the combined effects of AGEs and glucose shock pose a significant challenge to the normal beating function of the cardiomyocytes.

Shown in Fig. 10 is a graph that summarizes the dose dependent effect of AGEs on cardiomyocyte’s contractility. Statistical comparisons were made within groups. The control group exhibited a baseline beat rate of 62 beats per minute (BPM). Treatment with a low concentration of AGEs (100 µg/ml) significantly decreased beat rate to 56 BPM. Whereas a high concentration of AGEs (250 µg/ml) induced a further suppression, lowering the beat rate to 37 BPM. When the AGEs were combined with glucose at low concentration of AGEs (100 µg/ml), a modest increase in the beat rate to 63 BPM was observed compared to the control. This effect was amplified with a higher AGEs concentration (250 µg/ml), significantly increasing beat rate to 70 BPM. The addition of an AGEs inhibitor with the low concentration of AGEs (100 µg/ml) induced a reduction in the beat rate to 52 BPB. This inhibitory effect was further intensified with higher concentration of AGEs (250 µg/ml) that decreased to 41 BPM. Notably, when the AGEs inhibitors were combined with low concentration of AGEs (100 µg/ml) and glucose, the beat rate remained at 70 BPM. However, at the higher concentration of AGEs (250 µg/ml), this combination slightly mitigated the beat rate to 61 BPM compared to the effect of AGEs alone. The findings highlight the intricate relationship between AGEs and cardiomyocyte functions, demonstrated by alterations in the beat rate influenced by both AGEs and glucose levels.

Figure 11A presents brightfield imaging of iCell cardiomyocytes cultured under control conditions, showing contractile cardiomyocytes. These cells display clear nuclei and well-defined cell borders, indicating healthy morphology and structural integrity. Figure 11B demonstrates immunofluorescence staining of iCell cardiomyocytes, highlighting the intracellular localization of myosin heavy chain (MHC) using Alexa Fluor 488 conjugated antibodies. Figure 11C depicts the expression of MYH7, the gene encoding beta myosin heavy chain, involved in muscle contraction, in beating iCell cardiomyocytes. Quantitative PCR analysis showed a significant upregulation of MYH7 expression, indicating a 2.5-fold increase in MYH7 mRNA levels relative to GAPDH, a reference gene. Interestingly, the expression of Cx-43 was very moderate in these cells as depicted in Supplementary Fig. 4 . With cardiomyocytes differentiated from human iPSCs where they are at an early differentiation stage, the Cx-43 expression is not yet strongly upregulated. Enhanced expression of Cx-43 in iPSC-derived cardiomyocytes significantly improves intercellular coupling, modulates voltage-gated Na + channels (Nav1.5), and enhances electrical activity, suggesting Cx-43 as a novel target for enhancing iPSC-CM functionality to match native myocytes [1, 30]. Supplementary Video-1 depicts the normal cardiac beats per minute (BPM) for these cells.

## 4. Discussion

Elucidating the pathophysiological mechanisms behind diabetic cardiomyopathy still poses a challenge for researchers. One of the main reasons for this is the lack of accurate models, such as animal models, which can mimic the condition effectively. Additionally, studying both animal models and diabetic patients directly is extremely difficult due to various reasons. For instance, ethical considerations, patient consent, and the invasive nature of acquiring heart tissue. Even when heart tissue samples are obtained, they are mostly collected from patients in the end-stage of heart failure. Unfortunately, these patients often have other comorbidities in addition to diabetes, making it challenging to isolate the specific effects of diabetes on cardiac physiology [1, 31]. An immediate need arises for an in-vitro study model that surpasses these limitations, offering valuable insights into the development and impact of diabetes on cardiac physiology. In vitro study models are thus essential tools in diabetes research for dissecting the complex interactions between diabetes and cardiac physiology, enabling detailed mechanistic studies, and facilitating the development of effective treatments.

In this study, we aimed to investigate the effects of hyperglycemia (glucose shock) and advanced glycation end products (AGEs) on cardiomyocytes. This model allowed us to control and manipulate specific variables, such as glucose levels, and the presence of controlled doses of AGEs. This precise control helped isolate the effects of diabetes on cardiac cells without the confounding influences present in whole organisms. To do this a reduction strategy was employed to simulate a diabetic environment and evaluate the impact on a cellular level. Human AC16 cardiomyocytes, an immortalized cell line was used, which is commonly used to study signaling pathways involved in cardiomyogenesis. In our laboratory, we have used these cells extensively for modeling cardiac tissues as evidenced in numerous publications from our research [1, 32]. These cells are useful for evaluating the effects of drugs and developing therapeutic strategies for myopathological conditions [1, 33]. However, it is important to note that AC16 cardiomyocytes do not exhibit major inward or outward currents. Consequently, these cells do not beat or send electrical signals. On the other hand, human-induced pluripotent stem cell (iPSC)-derived cardiomyocytes were also utilized in this study as a robust cell type used to study cardiomyogenesis [1, 34]. These cells express fundamental cardiac ion channels [1, 35], allowing them to exhibit beating behavior and generate essential electrical currents necessary for functional cardiac contractility. The use of AGEs as an induced diseased environment in vitro was demonstrated by employing both AC16 cardiomyocytes and human iPSC-derived cardiomyocytes. This approach highlights the versatility of AGEs as a tool to study and understand the effects of diabetic conditions on cardiac functionality. Additionally, the study employed various environments to demonstrate the effects of AGEs on cardiac functionality. These environments included: AGEs (500 µg/ml), D-Glucose (50 mM), AGEs + D-Glucose, FPS-ZM1 (0.2 mM) + AGEs, and FPS-ZM1 (0.2 mM) + AGEs + D-Glucose. The various conditions were used to showcase distinct cell cultures and conditions, aiding in the comprehension of role of AGES in different scenarios.

Prior studies have primarily used animal models to investigate the effects of glucotoxicity on cardiac functionality in DCM. For example, one study utilized murine-based cardiac tissue models treated with different concentrations of AGEs, which resulted in the stimulation of markers of cardiac dysfunction [1, 36]. Other studies have also contributed to the understanding of glucotoxicity, in terms of hyperglycemia and AGEs, particularly in the preparation of AGEs. In this study, modified protocols from a previous study were used to prepare AGEs, but instead of focusing on renal cells [1, 16], cardiac cells were used. This connection to prior studies allows for the application of existing knowledge and techniques to investigate the effects of glucotoxicity on cardiac cells specifically. This study further advances the field of cardiovascular disease research by providing valuable insights into the effective use of AGEs dosage and effectively simulating a diabetic environment. By utilizing cardiac cells and focusing on the impact of glucotoxicity, this study contributes to a better understanding of the mechanisms underlying mechanisms of diabetic cardiomyopathy.

AGEs solutions were used in a concentration of 250 and 100 µg/ml each in this study. The guidance towards adopting these doses was obtained from others published works. Specifically in one study, AGEs (100 µg/ml for up to 90 mins) cause endothelial dysfunction in human coronary artery vascular endothelial cells through activation of p38 and ERK1/2 [1, 37, 38]. In another study, AGEs-induced autophagy in cardiomyocytes when a 100 µg/ml dose was applied to cells for 24-hrs [1, 39]. In our study, the 100 µg/ml dose of AGEs was not lethal to cells within 24 hours of exposure. Therefore, we systematically increased the dose in a stepwise manner to include three significantly different concentrations of AGEs to study their variable effects on cardiac cells in this study.

It was observed that AGEs possess a dose-dependent detrimental effect on the viability of AC16 cardiomyocytes. This exposure to AGEs not only led to the downregulation of the crucial cardiac gene GJA1 that encodes the cardiac gap junction Cx-43, but also induced significant alterations in the expression of the cardiac biomarker MHC, all of which are strongly associated with cardiac dysfunction [1, 40]. In the context of cardiomyogenesis in this study, our focus was mainly on GJA1/Cx-43 and MHC because GJA1/Cx-43 is crucial for forming gap junctions that facilitate electrical communication between cardiomyocytes, which is essential for synchronized heart contractions. Additionally, MHC, or Myosin Heavy Chain, is a major contractile protein in cardiac muscle, vital for the mechanical function of the heart. While other cardiac cell markers such as Troponins, Natriuretic peptides, Actinin, and Myosin are also important, GJA1/Cx-43 and MHC are directly involved in the fundamental electrical and mechanical properties of cardiomyocytes, making them key targets for understanding and improving cardiac function [1, 41]. Furthermore, the study unveiled the intricate relationship between AGEs, glucose shock, and cardiomyocyte contractility, suggesting a complex interplay between these factors and overall cardiac function. The findings were not limited to AC16 cardiomyocytes, as human induced pluripotent stem cell-derived cardiomyocytes also exhibited dose-dependent cytotoxicity and experienced changes in contractility upon exposure to AGEs. The higher the doses of AGEs administered the more pronounced the cell death observed. These findings shed light on the paramount importance of metabolic stressors in relation to heart health, particularly within the context of diabetes. It is evident that AGEs and high levels of glucose may play a pivotal role in the pathogenesis of cardiovascular complications associated with this chronic condition.

It is important to recognize that the study conducted utilized various cell types, each expressing different cardiac proteins and displaying distinct behaviors within the control group. Specifically, the AC16 cardiomyocytes exhibited a higher expression of Cx-43 but lacked contractile behavior. These cells do not express MHC, typically associated with healthy cardiac conditions. Instead of indicating a healthy state, the presence of MHC expression appears to be a response to stress induced by the various treatments added. This finding suggests that the AC16 cardiomyocytes cells may be more sensitive to environmental changes, reflecting their altered state rather than a baseline of normal cardiac function. AC16 CM cells are particularly valuable in tissue culture studies due to their unique composition. These cells are a fusion of cardiomyocytes and cardiac fibroblasts, which account for their specific expression profile. Notably, AC16 CM predominantly expresses connexin 43, a gap junction protein essential for intercellular communication in the heart. However, they exhibit lower expression levels of other cardiac biomarkers [1, 33] typically found in mature cardiomyocytes such as MHC. This selective expression pattern can be attributed to their hybrid nature, which influences their functional characteristics and response to stimuli. On the other hand, the iCell cardiomyocytes demonstrated enhanced expression of MHC, along with contractile behavior. The role of Cx-43 in the heart is crucial, as it is recognized as a key element in the propagation of electrical impulses, which is vital for proper cardiac function. However, it is worth noting that Cx-43 is also present in many cell types where the physiological propagation of electrical impulses does not occur. This is the case of the AC16 CMs, where Cx-43 might have additional physiological functions apart from electrical impulse propagation in comparison to the iPSCs derived CMs such as facilitating the exchange of molecules between adjacent cells or between cells and the extracellular space [1, 42]. The iCell cardiomyocytes did not exhibit clear expression of this exact connexin, suggesting the presence of other connexin proteins that contribute to electrical propagation. Regarding MHC, the expression was minimal to nonexistent within the control group of AC16 cardiomyocytes. Conversely, evident expression of MHC was observed in the iCell cardiomyocytes. This discrepancy can be attributed to the essential role MHC plays in powering heart contractions. By converting chemical energy into mechanical force, MHC acts as the molecular motor that drives the contraction of the heart [1, 43]. This phenomenon is visibly manifested as beating contractility. It is important to note that AC16 cardiomyocytes only displayed MHC in the presence of AGEs or hyperglycemia, indicating irregular or unusual expression levels of this protein. In light of these observations, it becomes imperative to conduct comprehensive investigations involving both cell types to obtain a holistic understanding of how various stressors may impact the heart. Such an approach will enable us to delve deeper into the complexities of cardiac function and develop more targeted interventions to safeguard heart health in the face of diverse metabolic challenges.

When AGEs levels are elevated, they can have significant consequences on cardiac function. These consequences include decreased myocyte contractility and increased myocyte apoptosis [30]. This observation is supported by other reports that the accumulation of AGEs is intricately linked to cardiovascular complications by causing mitochondrial dysfunction and oxidative stress. This can trigger proliferative, inflammatory, and fibrotic responses, potentially impairing cardiac electrophysiological characteristics [1, 44].

These effects were also confirmed in our studies compared to the control group, treatment with AGEs, AGEs + D-Glucose, FPS-ZM1 + AGEs, and FPS-ZM1 + AGEs + D-Glucose significantly altered RAGE expression in AC16 cardiomyocytes. Specifically, treatments with AGEs or D-Glucose alone slightly upregulated RAGE expression, while their combination resulted in a significant upregulation. The addition of the FPS-ZM1 with AGEs modified this expression profile, yet combining FPS-ZM1 with both AGEs and D-Glucose further increased RAGE expression. These results suggest that RAGE expression is upregulated in response to AGEs under hyperglycemic conditions and inflammation, highlighting the intricate interplay between these factors in the regulation of RAGE in cardiomyocytes.

Interestingly, although it is expected that cardiomyocytes express membrane-bound RAGE, our study revealed its absence. This indicates that the RAGE protein in the cells may have been internalized or translocated to the nucleus under hyperglycemic conditions, demonstrating its dynamic regulatory pathways [1, 45, 46]. This translocation could reveal a multifaceted mechanism involving mostly DNA repair mechanisms that help to mitigate hyperglycemia induced cellular damage. Or it could be mediated via RAGE translocation to the nucleus and significantly influencing the expression of genes related to the stress response. Finally, there could be involvement and activation of stress related transduction pathways, where RAGE signaling is involved [1, 47]. These findings collectively suggest that the role of AGES extends beyond membrane signaling to nuclear functions that might be crucial for cellular adaptation and survival under metabolic stress.

Our study aimed to investigate the impact of AGEs and glucose shock on various aspects of cardiomyocyte physiology, and this holds significant interest due to the increasing prevalence of diabetes and the corresponding rise in cardiac complications associated with the disease. Understanding and addressing the interplay between diabetes and cardiovascular health is crucial for developing effective prevention and treatment strategies.

On the other hand, prolonged and sustained hyperglycemia enhanced the cardiac beating rates in the iCell cardiomyocytes in our study. Increased glucose within the cells led to increased energy levels and more contraction. Prior published studies from others have reported that increased glucose metabolism via the GLUT1 receptor is indicative of cardiac regeneration [1, 48] and is often witnessed in neonatal cells and tissues. Since the cells used for the electrophysiology experiments were derived from iPSCs it can be estimated that these cells retain stemness and possess an advanced potential to regenerate in the event of an injury or disease. Thus, this cell type is a particularly useful tool for studying the development of diabetes in cardiac tissues in vitro. However, in actual diabetic cells, specifically GLUT-4 in adult cardiomyocytes, translocation to the membrane is limited due to insulin insensitivity. Therefore, these trends will need to be validated with more mature and patient derived cardiomyocytes obtained from diabetic patients [1, 49].

The utilization of defined culture conditions to induce diabetes, as demonstrated in this study, holds significant promise for examining the impact of diabetic metabolic stressors on cardiomyocytes, ultimately contributing to our understanding on how diabetes develops and affects cardiac physiology. To construct an accurate diabetic cardiac in-vitro study, it is crucial to identify the essential elements necessary for faithfully representing the cellular functional relationships relevant to DCM. The results generated from this study offer valuable insights into these relationships, laying the foundation for the development of a new model that can be employed to investigate the intricate cellular processes associated with cardiac dysfunction. Such an in-vitro model could facilitate drug screening for compounds targeting diabetic diseases and eventually bridging the gap toward patient-specific treatment.

The advantages of 3D in vitro growth compared to 2D monolayer cultures are widely recognized. In 2D cultures, cells are typically grown on a flat surface, which fails to accurately represent the complex three-dimensional structure and interactions found in living organisms. In contrast, 3D cell culture models more closely mimic the natural environment, allowing for the study of cellular behavior in a manner that better reflects the in vivo conditions [1, 50]. For this reason, future work involves the utilization of 3D bioprinting, which holds promise for the creation of functional cardiac tissue that closely mimics the complex 3D structure of cardiac tissue in vivo. This advancement will aim to replicate the intricate features of a diseased heart, more specifically DCM, by employing essential elements to accurately replicate its characteristics in an in-vitro model. By combining the capabilities of 3D bioprinting and the integration of disease-specific elements, as presented in this study, we hope to achieve a more accurate representation of diseased cardiac tissue for enhanced understanding and potential therapeutic applications.

## Conclusion

In this study, the successful utilization of in-house prepared AGEs to accurately simulate a diabetic environment, combined with hyperglycemic conditions, has been demonstrated. The research presented in this study offers a comprehensive exploration of the effects of AGEs and glucose shock on cardiomyocyte function. The study investigates various aspects of cardiomyocyte response, including cell viability, gene expression, and cardiac biomarkers, in both AC16 cardiomyocytes and human iPSC-derived cardiomyocytes. Through this analysis, dose-dependent cytotoxicity and alterations in contractility are revealed, highlighting the detrimental impact of AGEs on cardiomyocyte function. Notably, the study identifies a decrease and increase of essential cardiac biomarkers resulting in abnormal expression levels induced by AGEs, which could potentially contribute to cardiac dysfunction. The findings of this study, which elucidate the intricate relationships between AGEs, glucose levels, and cardiac function, have the potential to uncover new therapeutic targets for the treatment of heart failure.

Additionally, these findings may have practical applications in the field of cardiac bioengineering, opening possibilities for innovative approaches to address the underlying mechanism of diabetic cardiomyopathy.

## Supplementary Material

Supplement 1

## Figures and Tables

**Table 1:** Treatments, varying analyses, and cell presentations are summarized in a tabular format.

Step	AC16 Cardiomyocytes	iCell Cardiomyocytes
**Cell Treatment** *(Treat with different concentration of AGEs.)*	Control (0 μg/mL),100 μg/mL, 250 μg/mL, 500 μg/mL, for 24 and 48 hours at 37°C in a CO_2_ incubator	Control (0 μg/mL),100 μg/mL, 250 μg/mL, 500 μg/mL, for 30 minutes at 37°C in a CO_2_ incubator
**Cell viability Assay** *(Assess the cell viability using Live/Dead Assay)*	Stain cells with live/dead viability dyes. Analyze with fluorescence microscopy	
**Gene Expression Analysis** *(Evaluate the expression of GJA1 using qPCR)*	Use the comparative Ct method (2^-ΔΔCT^) to quantify the gene expression. Normalized the gene expression to GAPDH (endogenous control) for stability.	-
**Cytotoxicity Determination**	Calculate AGEs concentration that reduces cell viability approximately 50% LC_50_. Analyze data from live/dead assay results. Use dose response curve Analysis to calculate approximately LC_50_	-
**Immunostaining**	Visualize expression of MHC, Cx43, and RAGE	Visualize expression of MHC and Cx43
**Imaging**	Use fluorescence microscopy to capture images of cells from live/dead and immunostaining	
**Data Analysis**	Quantify live/dead cells, calculate approximately LC_50, a_ssess the expression levels of MHC, Cx43 and RAGE	Quantify live/dead cells, assess the expression levels of MHC, Cx43
**LEAP Assay** *(Evaluate electrophysiology characterization)*	-	Perform electrophysiology using standardized MEA techniques. Measure external field potentials inthe presence and absence of various biochemical treatments
**Time-lapse imaging** *(Monitor cardiac contractility)*	-	Capture the time lapse images of beating cardiac cells in the presence and absence of varying biochemical treatments and quantify the changes in the contractility parameters.

**Table 2:** The primer sequences corresponding to the genes for evaluation are shown below.

Gene mix primer	Forward	Reverse	Access gene No.
**GAPDH**	GTCTCCTCTGACTTCAACAGCG	ACCACCCTGTTGCTGTAGCCAA	NM_002046
**MYH7**	GGAGTTCACACGCCTCAAAGAG	TCCTCAGCATCTGCCAGGTTGT	NM_000257
**GJA1**	GGAGATGAGCAGTCTGCCTTTC	TGAGCCAGGTACAAGAGTGTGG	NM_000165

**Table 3:** Dose dependent effects of AGEs on percentage of living cells related to control.

AGEs Dosage	Percentage (%) of living cells related to control
**Control**	100
**100 μg/mL**	77
**250 μg/mL**	65
**500 μg/mL**	44

## Data Availability

The original contributions presented in the study are included in the article/supplementary material, further inquiries can be directed to the corresponding author/s.
