# Supplementary material for: Of cells and tissues: Identifying the elements of a diabetic cardiac in vitro study model
Source: Res Sq. 2024 Dec 20:rs.3.rs-5125697. Preprint. [Version 1] doi: 10.21203/rs.3.rs-5125697/v1 (PMC11702775; doi:10.21203/rs.3.rs-5125697/v1)
Supplement: Supplement 1 [file NIHPPRS5125697v1-supplement-1.pdf]

Supplementary Files

This is a list of supplementary files associated with this preprint. Click to download.

- GraphicalAbstract.jpg
- ControliCellCardiomyocytes1.avi
- SupplementaryInformation.pdf
